# Effects of Dietary Vitamin C Supplementation on Vitamin C Synthesis, Transport, and Egg Deposition in Breeding Geese

**DOI:** 10.3390/ani16010148

**Published:** 2026-01-05

**Authors:** Yanglei Hu, Rong Xu, Yating Zhou, Ning Li, Haiming Yang, Jian Wang, Hongchang Zhao, Jun Yu

**Affiliations:** 1 Department of Animal Science and Technology, Jiangsu Agri-Animal Husbandry Vocational College, Taizhou 225300, China; 19515908828@139.com (Y.H.); 19816528594@163.com (Y.Z.); tzwjian@126.com (J.W.); 18837101296@163.com (H.Z.); 2 College of Animal Science and Technology, Yangzhou University, Yangzhou 225009, China; a18852712451@163.com (R.X.); hmyang@yzu.edu.cn (H.Y.); 3 College of Animal Science and Technology, Shihezi University, Shihezi 832003, China; lining27y@163.com

**Keywords:** intestinal absorption, L-Gulonolactone oxidase, sodium-dependent vitamin C transporter, vitamin C deposition

## Abstract

This study aims to investigate the effects of dietary vitamin C supplementation on vitamin C synthesis, transport, and egg deposition in breeding geese. A total of 450 female and 90 male 221-day-old Yangzhou geese were randomly assigned to five treatment groups with six replicates each (15 females and 3 males per replicate). The control group received a basal diet, while the other four groups were fed diets supplemented with 100, 200, 300, and 400 mg/kg vitamin C over a 16-week feeding trial. The results showed that dietary vitamin C supplementation increased the vitamin C content in both serum and egg yolks and modulated the expression of key vitamin C-related genes. Specifically, the intestinal and ovarian sodium-dependent vitamin C transporters 1 and 2 (SVCT1/SVCT2) were upregulated, whereas hepatic and renal L-Gulonolactone oxidase (GLO) and SVCT1 were suppressed. These findings indicate that exogenous vitamin C enhances intestinal absorption, inhibits hepatic synthesis, and promotes yolk deposition, with 300 mg/kg emerging as an effective and practical supplementation level that provides a physiological basis for its application in poultry nutrition.

## Simple Summary

Vitamin C is an essential antioxidant that helps maintain the physiological balance in poultry. The nutrition of developing embryos, which relies on nutrients stored in the egg, significantly impacts poultry production. Unlike laying hens, geese have longer egg formation periods—approximately 10 to 12 days for yolk and about 3.5 to 4 h for albumen, which may favor vitamin C deposition in eggs. However, it has not yet been studied whether dietary vitamin C supplementation in breeding geese can effectively increase the vitamin C concentration in eggs. This study aims to investigate the effects of dietary vitamin C supplementation on vitamin C synthesis, transport, and egg deposition. The results demonstrated that dietary vitamin C elevated egg yolk and serum vitamin C levels, altered vitamin C transporter expression in intestines and ovaries, and suppressed synthesis-related enzymes in the liver and kidney. These findings indicate that exogenous vitamin C enhances intestinal absorption, inhibits hepatic synthesis, and promotes yolk deposition in geese.

## 1. Introduction

Vitamin C is a common natural water-soluble small-molecule compound that plays a crucial role in maintaining cellular redox balance by effectively scavenging reactive oxygen and nitrogen species [1,2]. In poultry, vitamin C can be acquired through endogenous synthesis, absorption from the diet, and renal reabsorption, which together ensure an adequate physiological supply [3,4]. Endogenous biosynthesis of vitamin C involves the reduction of D-glucuronic acid to L-guluronic acid, which cyclizes to L-gulonolactone and is then oxidized by L-gulonolactone oxidase (GLO) to form L-ascorbic acid [5]. Endogenous vitamin C synthesis may be down-regulated when dietary levels are sufficient, potentially reducing GLO activity [6,7,8,9]. Numerous studies have demonstrated that vitamin C supplementation improved the production performance of laying hens under stress conditions, including aging [8,10] and ambient temperatures [11,12,13,14].

The regulation of vitamin C uptake and its distribution in tissues is primarily managed by the action of sodium-dependent vitamin C transporters (SVCTs) [15]. Sodium-dependent vitamin C transporter 1 (SVCT1) is a low-affinity, high-capacity absorbable vitamin C transporter protein that is widely expressed in epithelial cells of various tissues, including the intestine, kidney, lungs, liver, and skin. It participates in systemic homeostasis and metabolic requirements by facilitating intestinal absorption and renal tubular reabsorption of vitamin C. In contrast, sodium-dependent vitamin C transporter 2 (SVCT2) is a high-affinity, low-capacity tissue transporter that is predominantly found in the brain, placenta, eyes, exocrine and endocrine tissues, and is responsible for the tissue transport of vitamin C [16]. The respective transport capacities and affinities for vitamin C are closely related to the recognized concept that SVCT1 mediates systemic vitamin C homeostasis, while SVCT2 ensures localized demand [17].

Domestic goose breeds exhibit a seasonal laying pattern regulated by natural changes in day length [18,19], and they are typically culled after 3 to 4 productive years. During the laying process, vitamin C is actively transported into the egg, which depletes maternal stores. Additionally, stress-induced corticosteroid synthesis increases the demand for vitamin C. Despite the physiological importance of vitamin C, research on whether vitamin C supplementation in diets can influence metabolism in geese and subsequently accumulate in goose eggs remains limited.

Currently, embryonic nutritional supplementation has significant potential to improve the performance of poultry production [20,21]. Unlike mammals, which transfer maternal nutrients and signals to the fetus through the placenta, avian embryos rely entirely on nutrients and maternal information stored within the egg [22]. These components depend on the nutritional and physiological condition of the breeder bird that laid the egg. In poultry production, the nutrient content of hatching eggs can be enhanced either by enriching the diet of breeders or through direct in ovo injection of specific nutrients [23]. The yolk and albumen of poultry are formed in the ovary and magnum, respectively [24]. Zhu et al. [9] found that dietary supplementation of 200 or 400 mg/kg of vitamin C to laying hen diets did not result in vitamin C deposition in eggs. However, geese differ from hens in egg formation dynamics: yolk development requires 10 to 12 days in geese compared with 7 to 10 days in layers, and albumen formation in the magnum lasts about 3.5 to 4 h in geese, nearly twice as long as the 1.5 h observed in chickens [24,25]. This longer formation period of goose eggs in both the ovary and the magnum may be more favorable for vitamin C deposition, which could benefit embryonic development by enhancing antioxidant status, supporting immune function, and promoting early growth in poultry [26,27,28].

Therefore, the objective of this study is to investigate the effects of dietary vitamin C supplementation on the vitamin C synthesis, transport, and egg deposition in breeding geese by assessing intestinal SVCT1 and SVCT2 expression to evaluate absorption, measuring serum vitamin C concentrations to assess systemic transport, examining ovarian and magnum SVCT1 and SVCT2 expression to elucidate vitamin C deposition in eggs, and analyzing hepatic and renal SVCT1, SVCT2, and GLO expression to investigate alterations in endogenous biosynthesis.

## 2. Materials and Methods

### 2.1. Birds and Experimental Design

This experiment was conducted at the National Waterfowl Gene Bank (Jiangsu), a branch facility of Jiangsu Agri-animal Husbandry Vocational College, where all experimental animals were sourced. A total of 540 221-day-old Yangzhou geese in good health, with consistent feeding level and in the egg-laying period, were selected as research subjects, and the male to female ratio was 1:5. The birds were randomly assigned to five treatments with six replicates each (15 female geese and 3 male geese per replicate). The control group received only a basal diet, and the other four groups were given diets supplemented with vitamin C at doses of 100, 200, 300, and 400 mg of vitamin C per kg of diet over a 16-week feeding trial. The vitamin C with a chemical purity of ≥95% was produced from Hangzhou Tiannong Bio-Nutrition Technology Co., Ltd. (Hangzhou, China). Batches of the experimental diets were produced every 4 weeks to limit the loss of vitamin C activity during feed storage. A basal diet was formulated to provide adequate concentrations of all the nutrients required by geese, according to the NRC (1994) [29] and prior research results [30] (Table 1).

The experimental animals were kept in a semi-open shed, with floor-based individual penning and flat rearing. Each goose was provided with 0.33 m^2^ of indoor enclosed space and 1.67 m^2^ of outdoor exercise area, including access to a small pond. The goose house was equipped with a trough, nipple water dispenser, an egg-laying box covered with soft straw. Throughout the experiment, the geese were exposed to natural light and ventilation with free access to feed and water. The housing facility was cleaned daily to maintain hygienic conditions.

### 2.2. Sample Collection and Preparations

One week before the end of the experiment, all hatching eggs were collected. Six eggs from each treatment were selected for analysis of the vitamin C content.

In the end, six female geese from each treatment were randomly selected for sample collection. Blood samples were taken from the wing vein between 8:00 and 8:30 a.m. and then centrifuged at 2000× *g* for 10 min to harvest serum, which was stored at −20 °C for further analyses. As the experiment had reached its predetermined endpoint, the selected geese were euthanized by cervical dislocation and subsequently exsanguinated. After bleeding, each goose was dissected, and the liver, kidney, reproductive tract (ovary and magnum), and intestinal mucosa (duodenum, jejunum, and ileum) were collected. All tissue samples were immediately flash-frozen in liquid nitrogen and stored at −70 °C for further analyses.

### 2.3. Measurement of Vitamin C Content in Serum and Produced Eggs

One milliliter of pre-cooled extracting solution and 0.1 g of yolk or albumen were added into a 2 mL centrifuge tube. The mixture was homogenized in an ice water bath, and then centrifuged at 8000× *g* and 4 °C for 20 min. The vitamin C content in the supernatant and serum was measured using commercially available kits (AIDISHENG, Yancheng, China), following the manufacturer’s instructions. Briefly, vitamin C was quantified using a microplate colorimetric assay, in which the reaction product forms a measurable chromogenic compound. Absorbance was measured at 534 nm using a Multiskan FC microplate photometer (Thermo Fisher Scientific, Waltham, MA, USA).

### 2.4. Quantitative Real-Time PCR

Total RNA was isolated from the liver, kidney, reproductive tract (ovary and magnum), and intestinal mucosa (duodenum, jejunum, and ileum) using TRIzol reagent (Tiangen Biochemical Technology Co., Ltd., Beijing, China). The quality and quantity of the RNA samples were verified, and cDNA was synthesized using Hifair III Reverse Transcriptase for quantitative PCR (Yeasen, Shanghai, China). SVCT1 and SVCT2 expression in the liver, kidney, reproductive tract, and intestinal mucosa, and GLO expression in the liver and kidney were analyzed using Hieff qPCR SYBR Green Master Mix (Yeasen, Shanghai, China) on the CFX96TM Real-Time System (BIO-RAD, Singapore). The detailed reaction system was referred to our previous description [31]. The primer sequences are presented in Table 2. The relative mRNA expressions were calculated using the 2^−ΔΔCt^ method, with β-actin as the housekeeping gene.

### 2.5. Statistical Analysis

All data were analyzed using one-way ANOVA, followed by Tukey’s post hoc test (SPSS version 26.0, SPSS, Inc., Chicago, IL, USA). The results were presented as the mean value and standard error of the means (SEM). Orthogonal polynomial contrasts were applied to test linear and quadratic responses to increasing dietary vitamin C supplementation. The significant differences between treatments were considered at *p* < 0.05 and trends at *p* < 0.1.

## 3. Results

### 3.1. The Concentration of Vitamin C in Breeding Eggs and Serum

As shown in Table 3, dietary supplementation with vitamin C influenced the vitamin C content in both egg yolks and serum (*p* < 0.05). Dietary vitamin C supplementation was associated with linear increases in vitamin C content in egg yolks (*p* < 0.05), with a significant increase observed at the 300 mg/kg supplementation level. Serum vitamin C content increased as linear and quadratic (*p* < 0.05) response to increasing dietary vitamin C supplementation. However, there were no significant effects of dietary vitamin C supplementation on the vitamin C content in egg albumen (*p* > 0.05).

### 3.2. Intestinal Expressions of SVCT1 and SVCT2 Related to Vitamin C Absorption

**Figure 1:**
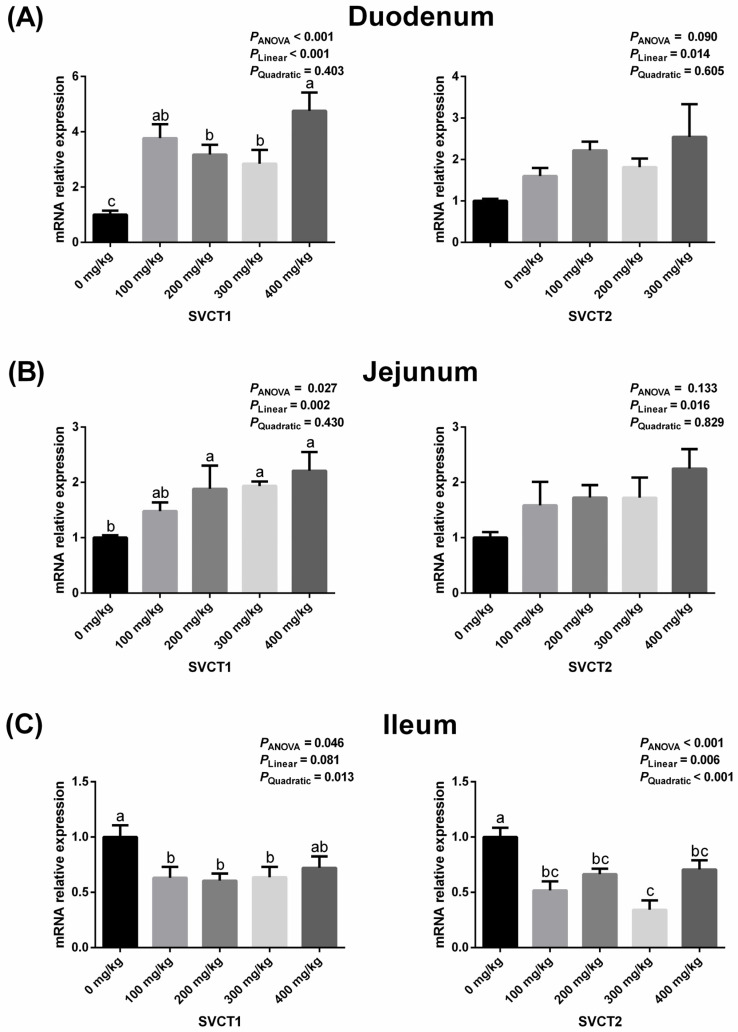
Effects of dietary vitamin C supplementation on the mRNA expression of SVCT1 and SVCT2 in the duodenum (**A**), jejunum (**B**) and ileum (**C**) of geese. Values are means ± SEM. ^a–c^ Means with different superscript letters are different (*p* < 0.05). SVCT1: sodium-dependent vitamin C transporter 1; SVCT2: sodium-dependent vitamin C transporter 2.

Dietary supplementation of vitamin C affected the expression of SVCT1 in the intestine and SVCT2 in the ileum (*p* < 0.05). As dietary vitamin C supplementation increased, the expression of SVCT1 increased linearly (Figure 1A, *p* < 0.05), while the expression of SVCT2 tended to increase (Figure 1A, *p* = 0.090) as a linear function (*p* < 0.05) in the duodenum. Moreover, dietary vitamin C supplementation was associated with linear increases in SVCT1 expression in the jejunum (Figure 1B, *p* < 0.05). However, dietary vitamin C supplementation led to a quadratic decrease in SVCT1 expression and both linear and quadratic decreases in SVCT2 expression in the ileum (Figure 1C, *p* < 0.05). There were no significant effects of dietary vitamin C supplementation on SVCT2 expression in the duodenum (Figure 1A, *p* > 0.05) and jejunum (Figure 1B, *p* > 0.05).

### 3.3. Ovarian and Magnum Expressions of SVCT1 and SVCT2 Related to Vitamin C Transport

**Figure 2:**
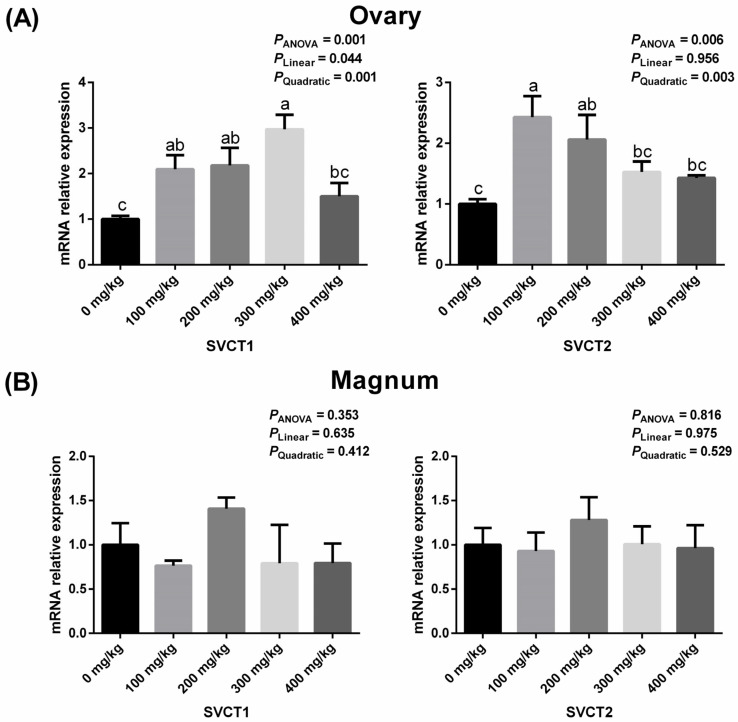
Effects of dietary vitamin C supplementation on the mRNA expression of SVCT1 and SVCT2 in the ovary (**A**) and magnum (**B**) of geese. Values are means ± SEM. ^a–c^ Means with different superscript letters are different (*p* < 0.05). SVCT1: sodium-dependent vitamin C transporter 1; SVCT2: sodium-dependent vitamin C transporter 2.

As shown in Figure 2, dietary vitamin C supplementation affected the expression of SVCT1 and SVCT2 in the ovary (*p* < 0.05). As dietary vitamin C supplementation increased, ovarian SVCT1 expression increased in both linear and quadratic manners, while SVCT2 expression increased linearly (Figure 2A, *p* > 0.05). However, dietary vitamin C supplementation had no significant effect on SVCT1 and SVCT2 expression in the magnum (Figure 2B, *p* > 0.05).

### 3.4. Hepatic and Renal Expressions of GLO, SVCT1, and SVCT2 Related to Vitamin C Synthesis

**Figure 3:**
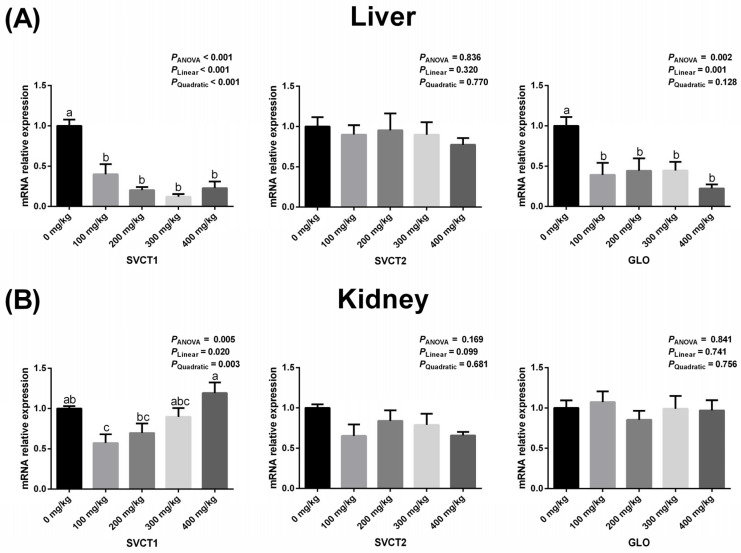
Effects of dietary vitamin C supplementation on the mRNA expression of GLO, SVCT1 and SVCT2 in the liver (**A**) and kidney (**B**) of geese. Values are means ± SEM. ^a–c^ Means with different superscript letters are different (*p* < 0.05). GLO: L-gulonolactone oxidase; SVCT1: sodium-dependent vitamin C transporter 1; SVCT2: sodium-dependent vitamin C transporter 2.

The effects of dietary vitamin C supplementation on the mRNA expression of GLO, SVCT1, and SVCT2 in the liver and kidney of geese are presented in Figure 3. In the liver, the expression of SVCT1 decreased both linearly and quadratically as the dietary vitamin C supplementation increased, while GLO expression exhibited a linear decrease (*p* < 0.05). In the kidney, SVCT1 expression decreased both linearly and quadratically in response to increasing dietary vitamin C supplementation (*p* < 0.05), with a significant reduction observed at the 100 mg/kg supplementation level (*p* < 0.05). However, dietary vitamin C supplementation did not have a significant effect on SVCT2 expression in either the liver or kidney, nor on GLO expression in the kidney (*p* > 0.05).

## 4. Discussion

Vitamin C is an essential micronutrient for poultry, known for its antioxidant and immune-modulating effects, which help reduce metabolic stress during reproduction [2,12]. Our previous study showed that dietary supplementation of 300 to 400 mg/kg vitamin C improved egg production, egg quality, and immune function in breeding geese [2], likely by alleviating oxidative stress during the laying stages. Therefore, this current study on this basis was to further evaluate the effects of dietary vitamin C supplementation on vitamin C synthesis, transport, and egg production.

In this study, along the small intestine from the duodenum to the ileum, dietary vitamin C supplementation first increased SVCT1 expression, and ultimately declined. SVCT2 expression exhibited an increasing trend in the duodenum, remained unchanged in the jejunum, and eventually decreased in the ileum. The higher expression of SVCT in the anterior small intestine indicates that dietary vitamin C supplementation promotes rapid absorption in this area. The subsequent decline in SVCT expression in the hindgut suggests a depletion of dietary vitamin C. Additionally, compared to vitamin C sourced from plant-based feed ingredients, additive vitamin C reaches the intestinal wall more rapidly and at earlier sites in the intestinal lumen, since it does not require digestion to be released from plant cells [32]. This finding may help explain why continuous dietary supplementation of vitamin C throughout the laying period in geese primarily enhances its absorption in the duodenum and jejunum rather than the ileum.

As dietary vitamin C enters the bloodstream, serum vitamin C levels increased in the vitamin C supplementation group in this study. This finding is consistent with Hooper et al. [6], who reported that dietary vitamin C supplementation can increase plasma vitamin C levels in chickens. However, it contrasts with Zhu et al. [9], who observed no significant effect of vitamin C supplementation on serum levels in Hy-Line Brown laying hens. This discrepancy may stem from the fact that geese in our study had continuous feed access, whereas the hens in Zhu et al.’s [9] experiment underwent a 12 h fasting period. Research indicates that after oral administration of high-dose vitamin C, it undergoes rapid metabolism and excretion within 3 h (even faster following intravenous injection), necessitating repeated dosing to maintain stable plasma vitamin C levels [33].

As a cofactor in collagen synthesis, vitamin C also promotes follicular growth in the ovaries. During follicular development, vitamin C accumulates in ovarian granulosa cells not due to de novo synthesis but as a result of increased expression of vitamin C transporters [34]. Gregoraszczuk et al. [35] found that at a concentration of 100 μM/L, vitamin C increased SVCT2 expression in human ovarian cells without affecting SVCT1 expression; whereas at a concentration of 1 mM/L, it increased both SVCT1 and SVCT2 expression in human ovarian cells. In the present study, dietary supplementation with vitamin C at doses of 100, 200, and 300 mg/kg resulted in increased SVCT1 expression, while supplementation at 100 and 200 mg/kg specifically increased SVCT2 expression in the ovaries. The enhanced expression of these transporters in the ovaries may facilitate the transport of vitamin C, leading to its effective accumulation in the yolk. This embryonic nutritional supplementation may have potential benefits for the growth performance of goslings after hatching.

In contrast, the expression of SVCT in the magnum remained unchanged, which suggests limited involvement of the transporters in albumen formation. Moreover, the short duration of albumen formation may further restrict vitamin C deposition in albumen [25].

L-gulonolactone oxidase (GLO) is a microsomal enzyme that plays a crucial role in the final step of vitamin C biosynthesis. Gene mutations in GLO can lead to the loss of vitamin C synthesis ability in certain species, such as humans [5]. In the present study, dietary supplementation with vitamin C reduced GLO expression in the liver, and a supplementation level of 100 mg/kg decreased GLO expression in the kidney, indicating that breeding geese possess an intrinsic feedback regulatory mechanism. These findings are consistent with earlier findings that dietary vitamin C supplementation reduces the need for endogenous synthesis by inhibiting hepatic GLO activity and expression in hens [6,7,8,9]. Since vitamin C absorbed in the small intestine first reaches the liver via the portal vein before being distributed throughout the body [34], it is likely that the liver serves as a sensitive indicator of vitamin C status.

In poultry, the biosynthesis of vitamin C primarily takes place in the kidneys and liver, with the kidneys producing significantly higher levels than the liver [7,8,9]. The minimal variation in GLO expression levels within the kidneys suggests that dietary supplementation of vitamin C has a little overall effect on endogenous vitamin C synthesis in geese. This underscores the importance of adding vitamin C to the diet during the laying period for optimal production. Based on the physiological and production responses observed, a supplementation level of 300 mg/kg appears to be both effective and practical, though further refinement is still necessary. Future studies could investigate vitamin C supplementation under various stress conditions or in geese raised for meat production to expand the applicability of these findings.

## 5. Conclusions

This study demonstrated that vitamin C supplementation modulates multiple aspects of vitamin C metabolism in breeding geese. Exogenous vitamin C suppresses hepatic synthesis via feedback inhibition of GLO, enhances intestinal absorption through upregulated SVCT1 and SVCT2 expression, and promotes yolk deposition by facilitating ovarian transport. A dietary supplementation dosage of 300 mg/kg appears to be both effective and practical, although further refinement is needed. These findings provide new insights into the metabolic regulation of vitamin C in geese and provide a physiological basis for its further application and investigation in poultry nutrition.

## Figures and Tables

**Table 1:** Composition and nutrient levels of the basal diet (air-dry basis).

Item	Content
Ingredient (%)	
Corn	64
Soybean meal	22
Rice husk	6
Wheat bran	0.5
Salt	0.3
Limestone	5.1
Calcium hydrogen phosphate	0.9
DL-Methionine	0.2
Premix ^1^	1
Total	100
Nutrient levels ^2^ (%)	
Metabolizable energy (MJ/kg)	11.08
Crude protein	14.74
Crude fiber	4.88
Calcium	2.17
Total phosphorus	0.49
Methionine	0.43
Lysine	0.74

^1^ Premixes provided per kilogram of diet: Vitamin A, 9000 IU; vitamin D3, 2000 IU; vitamin E 36 IU; vitamin K, 2.25 mg, vitamin B1, 2.2 mg; vitamin B2, 9.75 mg; vitamin B6, 3.75 mg; vitamin B12, 27.5 μg; vitamin B3 37.5 mg; folic acid, 1.4 mg; pantothenic, 15 mg; biotin, 95 μg; choline chloride, 550 mg; Fe (ferrous sulphate), 80 mg; Cu (copper sulphate), 5 mg; Mn (manganese sulphate), 100 mg; Zn (zinc sulphate); 100 mg; I (potassium iodide), 1.25 mg; Se (sodium selenite), 0.3 mg. ^2^ Crude protein, crude fiber, calcium, and total phosphorus were measured values, others were calculated values.

**Table 2:** Primer sequences for real-time PCR.

Gene Name	Primer Sequence (5′–3′)	Product Size (bp)	Accession Number
SVCT1	F: GTCCATCGTCCTCATCGTCC	133	XM_048064718.1
	R: GCCAGGATGATTGGGAACA		
SVCT2	F: CCAGGTTGTCATGTGCTCCT	124	XM_048053964.1
	R: GGCTGGAAGAAGTGGATCC		
GLO	F: CAGCGTCATCTACCAGGACC	150	XM_048079397.1
	R: AGAAGCGGTTGATCCAGCA		
β-actin	F: GCACCCAGCACGATGAAAAT	150	XM_013174886.1
	R: GACAATGGAGGGTCCGGATT		

GLO, L-gulonolactone oxidase; SVCT1, sodium-dependent vitamin C transporter 1; SVCT2, sodium-dependent vitamin C transporter 2.

**Table 3:** Effects of dietary vitamin C supplementation on vitamin C content in eggs and serum.

Item	0 mg/kg	100 mg/kg	200 mg/kg	300 mg/kg	400 mg/kg	SEM	*p*-Value		
							ANOVA	Linear	Quadratic
Yolk	11.83 ^b^	14.14 ^ab^	17.43 ^ab^	17.66 ^a^	17.46 ^ab^	0.719	0.019	0.0024	0.129
Albumen	12.56	13.48	14.13	17.12	15.60	0.802	0.421	0.099	0.708
Serum	1.38 ^c^	1.41 ^c^	2.02 ^b^	3.03 ^a^	1.93 ^b^	0.113	<0.001	<0.001	<0.001

Means within the same row that differ significantly are indicated by distinct superscript letters (*p* < 0.05). SEM: standard error of the mean.

## Data Availability

The original contributions presented in this study are included in the article. Further inquiries can be directed to the corresponding authors.

## Abbreviations

The following abbreviations are used in this manuscript:

GLO	L-gulonolactone oxidase
SEM	Standard error of the mean.
SVCT1	Sodium-dependent vitamin C transporter 1
SVCT2	Sodium-dependent vitamin C transporter 2
